# The involvement and autonomy of young children undergoing elective paediatric cardiac surgery: a qualitative study

**DOI:** 10.1186/s13019-022-01889-5

**Published:** 2022-05-31

**Authors:** Priscilla Alderson, Marc Cohen, Ben Davies, Martin J. Elliott, Mae Johnson, Alessandra Lotteria, Rosa Mendizabal, Emma Stockton, Michael Stylianou, Katy Sutcliffe, Hugo Wellesley

**Affiliations:** 1 Social Research Institute, University College London, 18 Woburn Square, London, WC1H 0NR UK; 2 Great Ormond Street Hospital for Children NHS Trust, London, UK; 3 Royal Children’s Hospital, Melbourne, Australia; 4 Cardiothoracic Surgery, University College London, London, UK; 5 Evelina Children’s Hospital, Guy’s and St Thomas’s NHS Trust, London, UK

**Keywords:** Cardiac surgery, Consent, Ethics, Paediatrics, Qualitative research

## Abstract

**Background:**

Standards generally reported in the literature about informing children and respecting their consent or refusal before elective heart surgery may differ from actual practice. This research aims to summarize the main themes in the literature about paediatric anaesthesia and compare these with research findings on how health professionals counsel young children before elective heart surgery, respect their consent or refusal, and maintain patient-centred care.

**Methods:**

This qualitative research involved: literature reviews about children’s consent to surgery and major interventions; observations of wards, clinics and medical meetings in two paediatric cardiology departments, October 2019 to February 2020; audio-recorded semi-structured interviews with 45 hospital staff, including 5 anaesthetists, and related experts, November 2019 to April 2021; interviews with 16 families, with children aged 6- to 15-years and their parents shortly after elective heart surgery, and some months later (reported in other papers); thematic data analysis; and research reports on how different professions contribute to children’s informed decisions for heart surgery.

**Results:**

The medical, ethics and English legal literature tend to assume legal minors cannot refuse major recommended treatment, and cannot consent until they are 12 years or older. Little is said about informing pre-competent children. If children resist, some anaesthetists rely on sedation and distraction, and avoid much informed discussion, aiming to reduce peri-operative anxiety. However, interviewees reported informing young children, and respecting their consent or refusal before elective surgery. They may delay elective surgery and provide further information and support, aiming to reduce fear and promote trust. Six years of age was commonly cited as the threshold for respecting consent to heart transplantation.

**Conclusion:**

Differing views about younger children’s competence, anxiety and best interests support different reactions to children’s consent and refusal before elective heart surgery. This paper reports the zero-restraint policy followed for over a decade in at least one leading surgery centre. The related law and literature need to be updated, to take more account of evidence of actual practice.

## Background

Consent to an invasive procedure mandates the discussion of benefits, risks and alternatives, and also implicitly the option to refuse treatment. When the courts consider cases of children who refuse major recommended treatment, they almost always authorize clinicians to proceed with the treatment [1]. A recent review of law in England and Wales concluded that, although judges listen carefully and compassionately to children’s views, they prioritise children’s welfare and best interests over their autonomy, by endorsing doctors’ decisions [2]. In law, interventions cannot be enforced on competent adults, but this may occur with children and is often “uncontested” [3, 4].

The anaesthesia mask or cannula is cited as children’s greatest peri-operative fear [5]. If they firmly resist before a non-emergency procedure, to which either they or their parents have previously consented, surgeons and anaesthetists have three options. Anaesthetists may restrain the child and proceed as quickly as possible. Second, they can try to forestall resistance by using stronger premedication and/or distraction [6]. Third, they can respect the refusal. “Cancellation of planned surgery because of child refusal is not uncommon,”[7] and is advised for older children [8]. We aimed to consider the benefits and harms of these three responses, and to question how they actually protect the child’s welfare and best interests.

In Britain, practitioners are advised to avoid conflict and favour negotiation, though the literature on consent and disagreements focuses principally on parents’ not children’s needs [9, 10]. The few papers on children and consent are about testing and managing them rather than listening to their views [9, 11], although the nursing and psychology press is more questioning about restraint [12–17]. In one survey, parents mainly reported their child’s physical problems relating to anaesthesia and children mainly reported their anxieties [18]. Clinicians are expected to provide developmentally appropriate information, to encourage children’s trust and respect their dignity [19]. Since the late 1980s, anaesthetists have been supported in these aims by the routine whereby parents remain with their child until the latter is unconscious, in the quiet anaesthetic room separate from the operating theatre [20], besides long-term policies of supporting parents to stay with their child [21]. Yet the parental presence is still vetoed and trialled in many countries [22] despite evidence that the parents’ presence significantly reduces anxiety [23].

Children’s consent is often termed “assent”, however this term lacks the history, meaning and legal status of consent and should not be mistaken as such. The child might not even be informed. Assent allows no choice, and contradicts English law [24, 25]. The Gillick law recognises the informed consent of the child aged under 16, with no stated minimum age, who “achieves sufficient understanding and intelligence to understand fully what is proposed [and has] sufficient discretion…to make a wise choice in his or her own interests” [26]. The law and literature tend to respect the consent of children from about 12 years upwards though not their refusal [27]. And little is said on younger children’s rights enshrined in the Convention on the Rights of the Child (UNCRC Article 12), ratified in every country except the USA: to be informed, to form and express their views, and to influence decisions when their views are “given due weight [by adults] in accordance with the age and maturity of the child” [28]. The emphasis is on UNCRC Article 3, “the best interests of the child shall be a primary consideration”, with “interests” being assumed to be defined by adults. To summarize, the general view in the law and literature is that legal minors may not refuse major treatment. The consent of “Gillick-competent” or “mature” minors may be valid. Pre-competent minors do not have legal rights to be informed or involved in decision-making.

This paper draws on research in two paediatric cardiac centres, which found that for over twelve years practitioners have been respecting young children’s consent and refusal. We report healthcare professionals’ views and experiences and consider why these differ from standards promoted generally in the literature.

## Methods

The qualitative research involved reviews of the law and literature on children’s consent to surgery and major interventions, and observations of wards, clinics and medical meetings in two paediatric cardiac surgery departments, from October 2019 to February 2020. With their written informed consent, audio-recorded semi-structured interviews were conducted with 45 hospital staff and related experts (see Table 1), from November 2019 to February 2020 face-to-face and, from March 2020 to April 2021 through contact by phone or online, because of COVID-19. Interviews were held with children aged 6- to 15-years and their parents shortly after elective heart surgery and six months later, a survey and group discussions (reported in other papers). Encrypted interview recordings were professionally transcribed and anonymised. Methods included thematic data analysis, multidisciplinary advisory group meetings, and regular research team meetings on analysing data and writing a series of papers [29].

**Table 1:** Specialties of the 45 interviewees

Specialty	Numbers of interviewees in each specialty. Some have two or three present or previous roles
Anaesthetists	5
Cardiologists	10
Chaplains	4
Children’s heart charities support and information services	5
Ethics committee members	8
Intensivists	2
Lawyers	3
Mediator	1
Members of hospital directorates	5
Nurses	6
Paediatricians (not cardiologists or anaesthetists)	6
Palliative care (paediatric)	1
Patient care coordinator	1
Play specialists	2
Psychologists	4
Psychiatrist and psychoanalyst	1
Senior lecturer in nursing	1
Senior Operating Department Practitioner	1
Social worker for heart transplant families	1
Surgeons	3

## Results

The literature and the interviews with professionals were analysed and compared regarding views on restraining children, sedating and distracting them, sharing information and discussion with them, and respecting children’s consent or refusal before elective cardiac surgery.

### Restraint

As noted, much of the literature on the law, ethics and clinical practice of children’s surgery assumes that the child’s refusal must be overridden, without mentioning that this may require physical restraint. To quickly impose the mask or cannula before surgery might seem to be the most cost-effective way to use expensive theatre time and other scarce resources, and to serve the child’s best interests by ensuring that essential surgery is provided. There is great pressure on clinicians to act in this way.

However, practitioners who were interviewed explained problems of restraining their patients. Anaesthetists wanted to avoid “harm from holding children down against their will. It takes away their control and it makes a lot of behavioural changes and psychological injury afterwards” (anaesthetist, interviewee number 1). “It’s the anaesthetists who are putting the brakes on…because we’re so exposed to the kids who have had that [coercion] done to them, who then come in terrified of everything” (anaesthetist, 18). These children then need much more time for their care than was saved by the initial restraint. Experiences of fearful helplessness can cause trauma that “can increase costs of care in the future”, and which may be prevented by sharing power, letting children know what to expect, encouraging resilience and recognising common fears and responses as normal [16, 30].

Children can be haunted by nightmares and lasting fearful memories [6]. These patients need life-long cardiac care, and interviewees were greatly concerned that they may become mistrustful, stop cooperating with their health-carers, and may stop attending clinics when they become adults.

### Premedication and distraction

Anaesthetists can reduce or avoid physical restraint when they use premedication and/or distraction. The aim is to reduce pre-operative anxiety, which can increase resistance and also correlates with “high postoperative pain, increased analgesic and anaesthetic consumption, and prolonged hospital stay” [31]. The case of a resisting 12-year-old who was deceived into taking ketamine was therefore approved by an ethicist [32]. Sedation can be highly effective. Oral midazolam can induce “that remarkable euphoric state of inebriation and reliable amnesia”, though it has drawbacks [33], and distraction may be more effective [34]. Distractions include pretending that the child is in a space rocket, hypnosis, “clowns, cartoons, magic tricks, and video games” [35, 36]. The effectiveness of distracting techniques and “behavioural interventions”^6 ^is assessed in trials that measure children’s anxiety levels by their observed and reported behaviours: “activity, vocalizations, emotional expressivity, state of apparent arousal” and parents’ observations [37, 38]. Provider-Tailored Intervention for Preoperative Stress (P-TIPS) assists adults to help “children to reframe a new, potentially frightening environment to something that is manageable and understandable… related to lower anxiety levels and increased coping behaviour”. Using P-TIPS, practitioners and parents can also be trained to encourage children’s ^“^coping-promoting behaviours”. Yet this involves “distraction, non-procedure-related talk, and humour”, and also discourages “distress-promoting behaviours”. The latter include procedure-related talk and “reassuring comments, apologies, criticism, empathic comments, or [giving] the child too much control over the medical procedure” [39]. Useful well-intentioned distractions have limitations. They rely on behaviourism, a set of beliefs summarised in Table 2). Anxiety felt by children in hospital is seen as a behavioural disorder, which is “unacceptable and preventable”[40] and to be controlled and suppressed with anxiolysis [41].

**Table 2:** Belief that anxiety before heart surgery is mainly physiological and behavioural

Anxiety is mainly:	Physiological, behavioural
It exists mainly in:	Behaviours, hormones
It is assessed by:	Measuring observed behaviours
It is best managed by:	Sedation, distraction
Emotions are:	Problems to be suppressed
Emotions such as anxiety:	Stop reasoned understanding

### Information and discussion

An alternative view (in Table3) is that anxiety before heart surgery is mainly social, connected to children’s awareness of the contexts, events and relationships. This view involves responding to children’s actual thoughts, needs, and reasonable self-protective anxieties before surgery. Children have been taught to fear strangers touching them and to say “no”. Many are afraid of being separated from their parents, of needles or scars, of being smothered, not waking up again, or dying [5, 16]. Uninformed children tend to fear they are being punished [42]. Whereas the first view manages the symptoms, anxious behaviours, the second view addresses causes of children’s anxiety through procedure-related talk. This may raise anxiety levels at first, but in order to help children to cope with their fears.

**Table 3:** Belief that anxiety before heart surgery is mainly social

Anxiety is mainly:	Social, emotional, reasonable
It exists mainly in:	Thoughts, feelings, relations
It is assessed by:	Listening to the child
It is best managed by:	Listening, explaining, reassuring
Emotions are:	Positive when trust and courage are nurtured
Emotions such as anxiety:	Are part of understanding pain and risk

Sedation and distraction may be used moderately along with discussions. “If I had somebody that was very, very scared about both [mask and cannula] I will give a pre-med” (anaesthetist 22). Yet pre-meds and distraction are used, not as in the first approach to replace supportive discussions, but to supplement them. And the aim is not to deceive children, as in the ketamine example [32], but to help them to relax and smile with their knowing agreement and cooperation.

Practitioners in the heart surgery centres carefully inform children as much as each child is able and willing to know. Before COVID-19, families could attend pre-operative interviews or clinics, now held online, and visit the operating and intensive care departments. Interviewees were asked the age when they would begin to inform children and respect their consent or refusal (reported in detail in another paper) [43]. On providing information, one anaesthetist said giving specific ages is too complicated and the four others said 3, 3, 4 and 6 years. An anaesthetist said:

As soon as they can talk to me basically. I’ll obviously tailor the conversation and the content, but when I go in the room the first person I talk to is the kid, not the adult...You can be three and know what “going to sleep” means even if you don’t know what an anaesthetic is…And even explaining post-op complications...“Your throat might feel a bit scratchy”(18).

Concern to inform very young children also applies to informing children with learning difficulties as fully and appropriately as possible, as we explain in another paper [44]. Interviewees aimed to balance giving information with managing anxiety. This anaesthetist’s views are typical.

Some people are reassured about knowing everything. Some people just get scared about knowing everything. [Yet they need answers to their worries.] Children can ask...about waking up [and] “Will I feel anything? Will I be aware when the surgeon cuts me? Will I be in pain?” Children are encouraged to get used to the mask, and to a doll with the lines and leads they will have, by playing with them. They are reassured lines are not inserted whilst they are still awake. And I say, “No, you are going to go to sleep first”...I normally say why we are doing this...“We’re going to make sure your heart is beating ok, your blood pressure is ok, and the rest of the body is working” [although]...If I can see a child who is getting more and more terrified I’ll just leave it there. [To avoid anxiety by withholding explanations can risk increasing it.] It’s scary to wake up and try and talk and there’s no voice coming out. [So children are warned about the tube that will need to be removed first.] Hyper-protective parents [want all information withheld so their child is not] upset. You need to keep the parents onboard...of course, they feel they know most and the best for their child [and you need]...to find a sort of common field. [Yet] the children are the patients still, so they need to be aware...you can’t trick them, they know what’s going on (22).

Working with parents may involve uncovering the parents’ own fears and bad memories (play specialist, 2). When they are helped to inform their child, “there’s been an uplift for the parents. You can see that they’re smiling, and they seem happier” (Advanced nurse practitioner 7). This takes time, and anaesthetists depend on nurses, psychologists and play specialists to prepare families thoroughly. The multidisciplinary team has to coordinate consistent information and nurture families’ trust in the whole clinical team. “If you’re not consistent then mistrust occurs and that’s far more difficult to come back from. Mistrust is an awful thing to have, is incredibly challenging…We need to work with families for the best outcomes and not battle through it” (Intensivist/anaesthetist, 13).

Children usually want to know what will happen while they are conscious during induction of anaesthesia, or what intensive care will be like, and some will want to know about surgery outcomes. Children are less interested in surgical techniques, though young children could be highly informed. A surgeon (19) recalled “a 7-year-old child…looking at his iPad and he was watching the operation I was going to do, he’d found it on YouTube, sitting there saying, ‘Is this what you're going to do?’”.

Instead of the unrealistic belief that patients’ anxiety is “unacceptable and preventable”,^10^ honesty with reassurance is the general approach.

You can’t take it [anxiety] away altogether but no one needs to have a terrible experience…You’ll get very sensible 5-year-olds that will say, “Could I die?”…I always answer their questions as truthfully as I can...I’d say, “Yes, you could do…You’re very clever that you’re asking that question because that’s a really sensible thing to be worried about. I will do everything I can to keep you safe and I’ll be there through the whole anaesthetic. I’ll never not be with you. I’ll be looking after you all the time. I think it’s extremely unlikely that anything bad will happen but there’s always a chance that something could happen…”(1).

During their brief pre-operation encounters with families, anaesthetists “have to be so dynamic. Lay people often do not know we are doctors. We have to explain so much, pick up their fears, the worst fear about surgery might be, ‘Will I wake up afterwards?’”(8). Adults are less likely to recall information, or regard the consent discussions about anaesthesia as important, or recognize them as a consent process, compared with their discussions about surgery [45]. Parents and children have difficulties with recalling information [46], and may best remember answers to their main concerns.

### Respecting children’s informed consent or refusal

Practitioners who talk with children about their anxieties and needs are more able to share informed decision-making with them, the basis of consent. The two views on the nature of anxiety (Tables 2 and 3) connect to different views about children’s best interests and the ethics and law of children’s consent (Table 4). Consent law protects adults from deception and coercion, but the first view (in column 2) assumes young children cannot make reasoned decisions and therefore may need to be controlled by adults in their best interests. The second view (column 3) assumes it is possible to reason with young children and, in the words of one of the interviewees, “to place *both* the physical and psychological wellbeing of the child above all else” [47].

**Table 4:** Two sets of beliefs about the ethics of responding to children’s anxiety before elective surgery

Anxiety is mainly:	Physiological, behavioural	Social, emotional, reasonable
If children resist, doctors should:	Continue interventions as quickly as possible	Allow time to negotiate
Coercion and deception are:	Necessary if in child’s best interests	Not in child’s best interests
Children’s best interests are:	To have surgery	To have surgery they can understand and cooperate with as much as possible
Adults should:	Be in control	Inform, involve and support children as much as possible
Laws on adult patients’ consent are:	Irrelevant to young children	Important guides and standards

Interviewees tended to say age is not the main criterion for assessing competence; more depends on children’s views, abilities, experiences and relationships. For example, an anaesthetist said: “No it’s definitely not an age only…some children are…happy with their parents’ decision, others more questioning”(22). Whereas the law and ethics literature generally set minimum ages of 12 years upwards for consent and 18 for refusal of major recommended treatment, interviewees tended to set younger ages: “7-, 8-year-olds can be very involved…[not] as a sole decision maker, but you certainly can be involving them in the decision” (anaesthetist 18). “It is easier for a 7-year-old to consent when told, ‘We are going to make you feel so much less breathless, you are going to have so much more energy, like you know to play football’ [than for those who are] asymptomatic because that’s the whole point of catching them before they deteriorate” (paediatric cardiologist 14). One 7- or 8-year-old “was so brilliant, he knew it all…what was going on…why he needed” the heart transplant because of long-term heart failure, after having “been around the hospital for years”. He knew “how rare it is to get a heart when you are a child” and that “it was the only way…not to die essentially…they feel like they’re gifted” (Anaesthetist 22). Adults’ and children’s consent is partly validated by the doctors’ integrity and honesty about recommended surgery. One anaesthetist spoke of “good governance” and “collegiate” decision-making in medical meetings, where 20 or more senior staff debate each elective case before they decide on recommended surgery (1).

On the age when they would begin to respect the consent of some children, the anaesthetists replied: 6- and 12-years. Another said, “Hyper-intelligent children who have had long illnesses and are at the centre of decision making, their age could be in single figures”(8). A fourth said, “You can have a 5-year-old who is much more sensible than a 14-year-old, independent of learning difficulties…[who can think] ‘Yes, we need it’…Even if it’s high risk, they will be onboard”(1). One did not give a specific age of consent but stated a quite widely held view:

About 6-years and above, they all understand to a certain level what’s going to happen…if the information is given to them…maybe they don’t know the details…what the defect is called or where the hole is and why it’s there. They know there’s something not quite right with the heart that needs to be fixed because they are either going to get worse or they already feel the symptoms are there and they need to have the surgery (22).

Contrary to much of the literature, most of the 45 interviewees said they respected children’s refusal at a lower age than they respected children’s consent, in terms of listening and avoiding force and deception [48]. Asked what he would do if a child were to firmly refuse anaesthesia before a non-urgent heart operation, a surgeon (15) replied,

Cancel it…We have a zero restraint policy so we wouldn’t ever tolerate the actual scenario where we’re restraining a children to have an anaesthetic...[The policy] was a pioneer for the UK…at least ten years ago...And I can certainly recall…people being held down…So it’s really frowned upon now…Anaesthetists are sort of perioperative advocates for patients and their families. So they are there at the beginning, the middle and end for any operation...or in a cath lab...One of their important roles is to act as an advocate and that includes psychological wellbeing of the child.

An anaesthetist considered: “Unless it’s a life-threatening emergency, you stop [the operation] and they go away and they think about it…the psychology team…can bring that child back with a plan in place that they’ve agreed to” (13). They may use POEMS [49]. **P**ositive **O**utcome and **E**xperience **M**anagement **S**trategies train professionals to talk with anxious children, work out with them why they refuse and how they can be helped, and may provide weekly play sessions to help them overcome their fears (Play specialist 2).

This help might seem to deny respect for children’s refusal. However, many children (and adults) consent to the agreed aims/ends of surgery: better health or prevention of future problems. Yet they greatly fear the means, such as masks and needles. To help them cope with their fears involves supporting them to achieve their main choice. When asked the age when they would begin to respect refusal by delaying surgery, three anaesthetists did not state a specific age, the others said 2-years and 4-years.

Occasionally, a child’s refusal is fully accepted. With heart transplants, the most cited threshold age was 6-years, when children are not added to the waiting list unless they are informed and “committed” (Senior psychologist 5). Respecting a 6-year-old’s refusal, when she “definitely didn’t want [a heart transplant, creates conflict] within a family because the parents don’t want to lose their child. [Yet] the compliance of the child is so pivotal, that if they are not onboard with it then you’ve wasted a huge resource” (Clinical nurse manager 11). Children may not long survive an unwanted transplantation, for example when they refuse daily immuno-suppression. The English courts last supported an enforced transplant in 1999 [50], and since then doctors have avoided requesting this [51]. These children’s names remain on a list in case subsequently they change their mind. No one mentioned complaints from parents about delays. Part of this process of trusting and listening to children involved informing, respecting and working with parents to serve the child’s best interests.^47^

## Conclusion

Differing views about younger children’s competence, about the nature of anxiety and the child’s best interests support different reactions to children’s refusal before elective heart surgery.

The law and literature generally support overriding children’s refusal. Yet practitioners who were observed and interviewed respect refusal. They delay elective surgery and provide further information and support, aiming to reduce fear and promote trust.

This paper is important because it reports practitioners’ respect for young children’s consent or refusal, practised for over a decade. The related law and literature need to be updated, to take more account of evidence of actual practice.

## Data Availability

The interview transcripts and observation notes, though anonymised, include personal details that could reveal individuals’ identity and in agreement with the research ethics authorities will therefore not be publicized.
